# Strategy maintenance in smart healthcare systems

**DOI:** 10.1186/s12911-023-02291-4

**Published:** 2023-11-28

**Authors:** Abir Boujelben, Ikram Amous

**Affiliations:** MIRACL laboratory, University of Sfax, Sfax, Tunisia

**Keywords:** Verification, Healthcare strategy, Anomalies, Healthcare system, Ontology

## Abstract

**Backgrounds:**

The size of medical strategies is expected to grow in conjunction with the expansion of modern diseases’ complexity. When a strategy includes more than ten statements, its manual management becomes very challenging, and in some cases, impossible. As a result, the updates they get may result in the unavoidable appearance of anomalies. This causes an interruption in the outflow of imperfect knowledge.

**Methods:**

In this paper, we propose an approach called TAnom-HS to verify healthcare strategies. We focus on the management and maintenance, in a convenient and automatic way, of a large strategy to guarantee knowledge accuracy and enhance the efficiency of the inference process in healthcare systems.

**Results:**

We developed a prototype of our proposal and we applied it on some cases from the BioPortal repository. The evaluation of both steps of TAnom-HS proved the efficiency of our proposal.

**Conclusion:**

To increase ontologies expressiveness, a set of rules called strategy is added to it. TAnom-HS is a two-step approach that treats anomalies in healthcare strategies. Such a task helps to take automatic and efficient healthcare decisions.

## Backgrounds

**Fig. 1:**
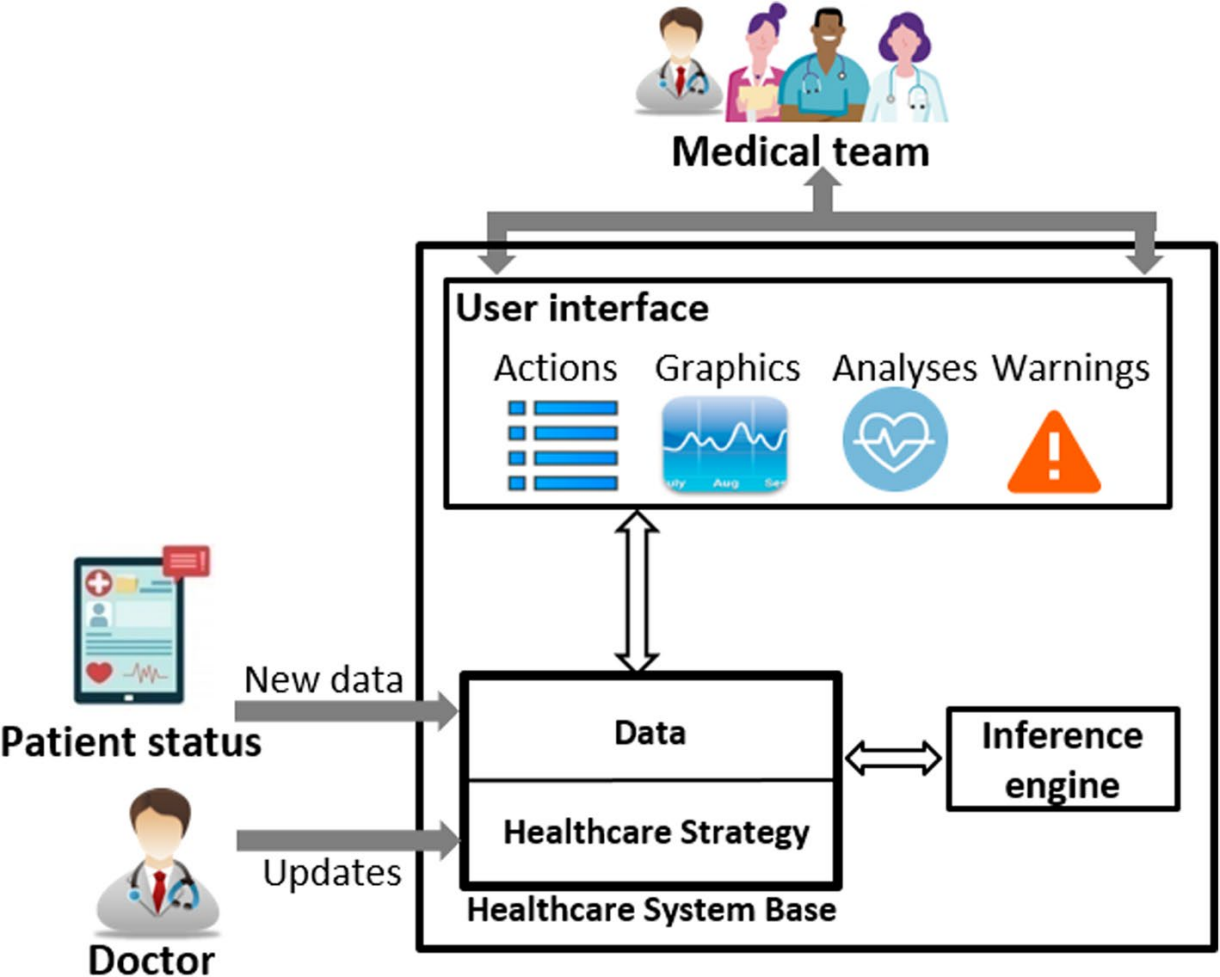
Smart healthcare system

In recent decades, improvements in medical care and computer technology have enlarged the traditional scope of medical services. This situation has opened up new possibilities for developing software to deliver enterprise services in a productive, varied, and highly dynamic environment. However, the complexity of IT-based healthcare systems is a result of the interaction of numerous components, including a variety of specialists and embedded devices (see Fig. 1). Such systems rely on services and use ontologies to manage complexity and heterogeneity. These services are supervised using a set of rules, called statements, associated to an ontology. A set of statements, called strategy, specify the appropriate course of action for every conceivable circumstance. And due to the huge sizes of healthcare strategies, their evolution causes the appearance of anomalies such as conflicting and redundant statements. These disruptions in the stabilized state of healthcare strategies have harmful effects on assets with erroneous decisions and treatments that are not adequate for the current situations. Take the example of an elderly hypertensive person who lives alone. In the case of a sudden rise in blood pressure, some data are quickly collected and analyzed. Then well-defined urgent treatments must be sent to a specific device, or a medical team must be notified for urgent intervention. If the healthcare strategy, in this case, includes some anomalies, the choice of decisions will be wrong. This will have a bad influence on the patient’s condition. It can even cause death. For such reasons, healthcare strategies must always be consistent.

In this paper, we present TAnom-HS an approach that ensures resolving anomalies that can affect a healthcare strategy. It includes two main steps: (I) extracting relations between statements and (ii) resolving anomalies. Our proposal includes a new technique for extracting relations between statements. As a result, the evaluation’s findings were enhanced in comparison to earlier studies that were focused on the same issue. During the anomalies processing step, we suggested handling some unconsidered anomalies. Moreover, we proposed an approach that made it possible to optimize treatments.

TAnom-HS acts also to help doctors and their teams to have a clear idea about the current situation of a patient in order to choose the right actions to take. These experts can ask for more details about the situation. The answers to these queries are provided by processing available data using the healthcare strategy.

In the rest of this paper, we present the state of the art about healthcare strategies then about anomalies and their resolution. After that, we exhibit the existing work about maintaining strategies. Afterward, we introduce our approach and its modules. We explain each step and we enlighten our contributions. After that, we present the evaluation of extracting relations between statements and the evaluation of resolving anomalies. At the end, we present the conclusion and our perspectives.

### Strategies and statements

An ontology is composed of a set of classes interconnected by relationships called object properties. A class also has relationships to data called data properties. Ontologies focus on classification techniques, especially the definition of «classes», «subclasses», how specific resources might be connected to such classes, and describing the connections between classes and their instances. The problem of ontologies’ lack of expressivity in the association and operation of properties to assist the reasoning of spatial relationships can be resolved by using Statements (also called inference rules)[1]. Statements focus on defining a general mechanism for identifying and creating new relationships based on current ones. Statements are the policies that underpin the deduction, derivation, or demonstration process. A healthcare strategy, composed of statements, makes it possible to describe the relationships that can exist between the different objects of an intelligent healthcare system.

**Fig. 2:**
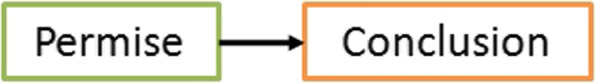
General form of a statement

In literature, a statement is defined as a function that takes formulas as input and returns a new formula [2]. The input part is called premise and the output part is called conclusion (see Fig. 2). The premise contains combined conditions using the AND logical operator. The conclusion part uses this same operator to combine conclusions (or actions). If all conditions are verified (or triggered), the conclusions are applied.

According to statement S40, a smart wearable blood pressure device detecting a systolic arterial pressure more than 180mmHg and a diastolic blood pressure more than 110mmHg should declare a hypertensive emergency.

$$\begin{aligned}{} & {} \mathbf {S40\!:}\ systolicArterialPressure(x,sap), equalOrGreaterThan(sap,180) ,\\{} & {} diastolicBloodPressure(x,dbp) , equalOrGreaterThan(dbp,110) \\{} & {} \longrightarrow HypertensiveEmergency(x) \end{aligned}$$

Each part of a statement is composed of a positive conjunction of tuples called atoms. Atoms may have different types such as object (e.g. Patient(x)), relation (e.g. wearsDevice(x,d)) and property (e.g. age(x,47)).

#### Relationships between statements

**Fig. 3:**
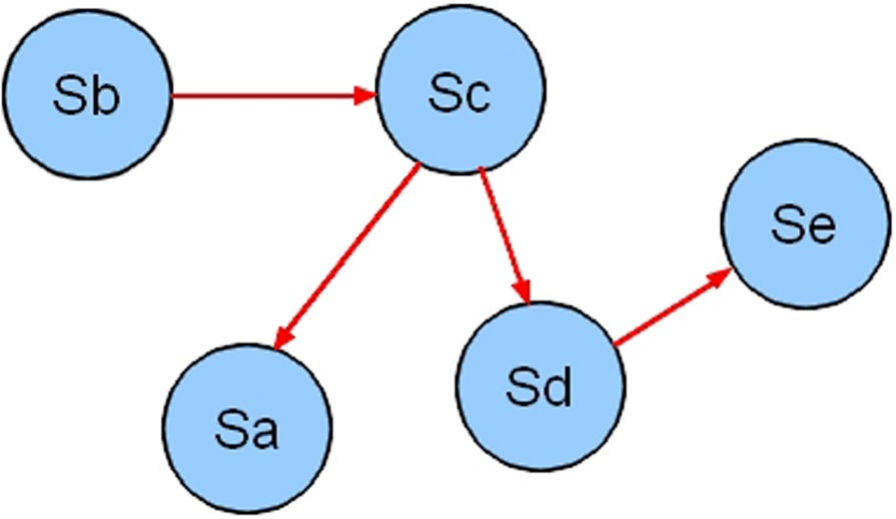
Example relationships graph

A relationship graph is defined as an abstract mathematical structure for representing complex causal dependencies between its vertices. In our case of study, a graph of relationships among statements is an oriented graph which vertices represent statements and the arcs represent the dependencies between these statements (see Fig. 3). An arc (A,B) of this graph indicates that the achievement/existence of A depends on the achievement/existence of B.

Relationships between strategy’s elements provide important insights. They can reflect the state of the strategy. This can be used in particular strategies management and in detecting anomalies . A relationship between two statements indicates that the second statement depends on the first one. It also shows that the first statement must be executed before the second. This implies that the application of the second statement requires the use of facts inferred by the first.

Extracting relationships between statements is an essential task in dealing with issues related to strategies: statements representation, generating the statements execution order, detecting anomalies, verifying explanations and answering queries.

In literature, the search for relationships between statements is carried out in different ways. Some methods refer to a criterion [3–5]. Others analyze the strategy’s usage data [6–9]. Other methods are based on extracting the relationships between the atoms of the premise and the atoms of the conclusion of each pair of statement in the strategy [10–14].

On the one hand, relationship analysis based on the analysis of the usage data requires a large number of iterations. This should be followed by an analysis of the consistency and accuracy of the results obtained after the iterations.

On the other hand, methods based on the analysis of atoms are based on verifying the existence of a relationship between each pair of statements. This amounts to checking whether the first statement can produce facts that can be used by the second. These methods are distinguished from other ones by the fact that they are applied independently of the execution process. So, this does not disturb the state of a running system. Moreover, these methods are based on the semantics expressed by the relationships between the objects. So, they are based on the logic of the medical field. Their main drawback is the fact that the analysis is based only on some types of atoms. In addition, we have noticed that the interfaces used for the representation of relationships are difficult to manage and to understand due to the large number of statements. The sizes of the obtained graphs are enormous. This results in an inability to interpret the detected relationships.

**Fig. 4:**
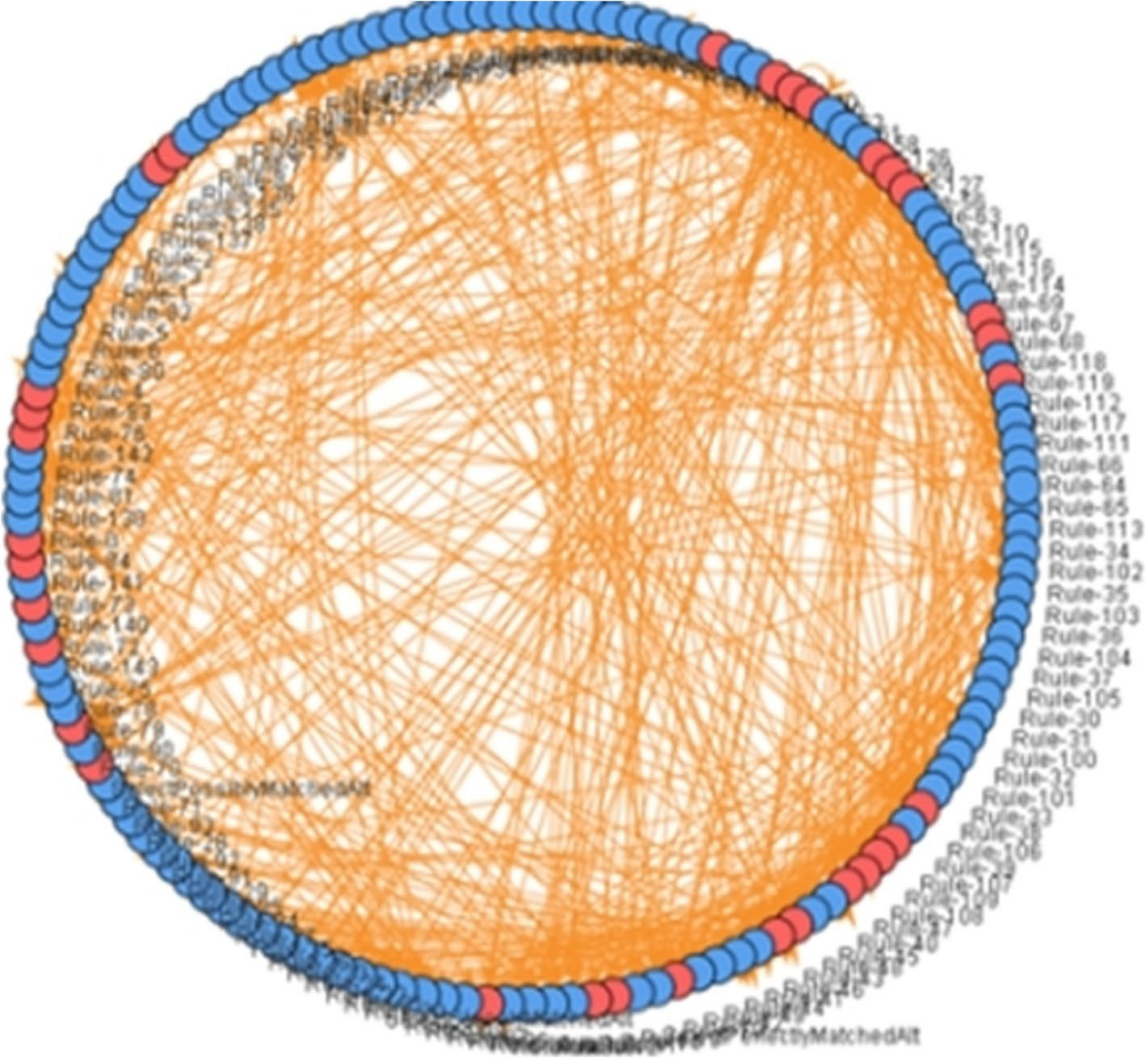
Relationship graph of the FHHO strategy

For example, Fig. 4 shows the relationship graph between the elements of the FHHO’s^1^ strategy that has approximately 200 items. It is obvious that understanding and managing this graph is too difficult.

### Dealing with anomalies

Works in literature classify anomalies (or also called potential problems) into two main categories: incompleteness and inconsistencies. A strategy is said to be complete only if all the inferred knowledge can be predicted by the medical team. A strategy is said to be incomplete due to inaccessible statements and illegal data, while inconsistency involves redundancies, conflicts, circularity and unnecessary conditions.

#### Incomplete Strategy

An incomplete strategy may include inaccessible statements and/or statements with properties with illegal values.

- Inaccessible statements: the inaccessibility of a statement comes from an unsatisfactory premise or from an unsatisfactory conclusion [15] (see Example 1).
  ***Example 1.*** Statement S30 is not accessible because its premise is unsatisfiable.
$${\textbf {S30 : }} DiabetesType1(x), DiabetesType2(x) \longrightarrow gets(x, Metformin)$$
- Illegal values of properties: these are values that violate the definition of their properties (e.g., a negative value for a patient’s age).

#### Redundancy

Redundancy leads to different types of problems: the consumption of superfluous memory and the consumption of time during inferences or even during updates [16]. Redundancy can exist in several forms: equivalence (or also called in some works equality), subsumption, and transitivity.

- Equivalence: two statements are said to be equivalent if they take the same entries and produce the same result. Example 2 presents an example of two equivalent statements.
  ***Example 2.***
$${\textbf {S01 :}} Patient(x), hasFood(x,y), {\textbf {hasIngredient(y,z)}} \longrightarrow eats(x,z)$$
  $${\textbf {S16 :}} Patient(x), hasFood(x,y), {\textbf {contains(y,z)}} \longrightarrow eats(x,z)$$
- Subsumption: There is a subsumption between two statements if they have the same conclusion, while the premise of one of them subsumes the premise of the other. Example 3 presents a case of subsumption.
  ***Example 3.***
  $${\textbf {S01 :}} Patient(x), {\textbf {hasFood(x,y), hasIngredient(y,z)}} \longrightarrow eats(x,z)$$
  $${\textbf {S17 :}} {\textbf {hasFood(x,y), hasIngredient(y,z)}} \longrightarrow eats(x,z)$$
- Transitivity: A redundancy of transitivity exists if a statement can be deduced from two or more other statements (see Example 6).

#### Circularity

**Fig. 5:**
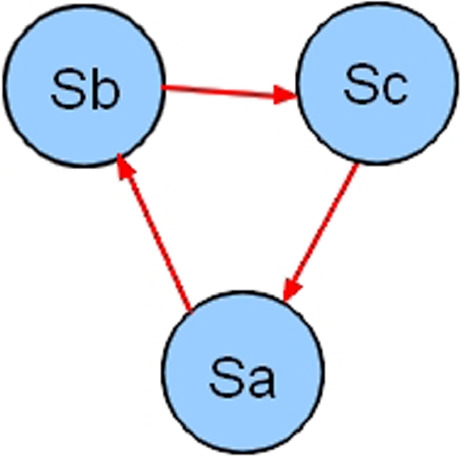
Cycle example

A cycle (also called infinite loop) is a set of statements that run endlessly [17, 18] (see Fig. 5). The existence of a cycle leads to an infinite inference process. Moreover, the knowledge involved is definitely lost [18].

#### Conflict

Some works define a conflict by the existence of two statements taking the same inputs and returning contradictory results [8, 19]. It is obvious that the conflicts must be resolved since they can lead to blocking the system or to erroneous knowledge. Take the example of two statements, one of which has the form $a \longrightarrow b$ and the other has the form $a \longrightarrow \lnot b$ (see S10 and S13 in Example 8).

### Maintenance of strategies

Currently, there are a variety of tools that allow manipulation and reasoning on ontologies. But a majority of them do not support strategies (e.g. FACT++ [20] and Snorocket [21]). In our work, we are interested in reasoning on strategies associated to ontologies. Some tools allowing it are presented in Table 1 (OntoStudio [22], LPA Visirule [23], Axiomé [13], SWRL Tab [24], TRANSLATOR [25], CLIPS [26], Drools [27]^2^). There are visualization and exploration tools, editors, and execution tools. Each of these tools has a set of tasks to perform. Some tools present a combination of these three categories. They are called BRMS for Business Rules Management Systems. Our study of surveys in literature [28–30] has allowed us to notice that:

- Most of the existing tools do not verify strategies and they are limited to ontologies (databases) verification.
- There are very few reasoners available that provide full reasoning support for the most recent semantic web languages, such as OWL-S and SWRL.
- Better management of strategies can be ensured by offering:
  - an understandable representation of the whole strategy or of each statement. The graphical representation helps enormously to facilitate this task.
  - an easy statements editing. It is a fundamental service since the environments of intelligent systems are scalable and the updating of knowledge bases is then imposed.
  - the verification and optimization of strategies. They are also required services since anomalies in strategies may the system to crash and infer erroneous knowledge.

**Table 1:** Reasoning tools supporting reasoning on statements

	Representation			Verification
	each statement	all strategy	graphic	
**OntoStudio**	**X**		**X**	**X**
LPA Visirule	X		X	
**Axiomé**	**X**	**X**	**X**	
SWRL Tab	X	X		X
**TRANSLATOR**	**X**			
CLIPS			X	
**Drools**	**X**	**X**		

In our work, we try to ensure these functionalities by offering a clear representation of the strategy and by eliminating the anomalies it may contain.

In literature, as in the existing tools, strategies verification is generally accomplished in two steps: (i) anomalies detection and (ii) their resolution. These two tasks were performed according to several methods.

- **Closure computing:** Yunchuan et al. [31] propose a method for eliminating redundancies, conflicts and cycles from a strategy. This work is based on the calculation of the Closure of the strategy. This amounts to adding all the statements that may be implied by the already existing statements. The anomaly detection is then performed using literal comparison.
- **Computation of the matrices of similarity:** Cheng et al. [32] introduce a method to solve the errors in knowledge based on statements. They use similarity matrices between the premise parts and similarity matrices between the conclusion parts. This technique has also been incorporated into other work dealing with the same issue, such as Sun et al. [33] who suggest detecting conflict through the analysis of the premise and conclusion parts. This work allows anomaly detection based on domain knowledge and probability analysis (e.g. probability of temperature increase). The process is done during system creation and running.
- **Methods based on the definition of meta-models:** Aloulou et al. [34] propose an approach to resolve anomalies in order to facilitate the creation of information systems in the context of ambient intelligence. The major contribution of this approach lies in its ability to detect unsatisfactory statements and conflicting statements. Hassanpour et al. [13] propose a method that allows defining patterns for the elements of the database which allowed to put them in groups to facilitate the exploration of the entire strategy.
- **Methods based on the analysis of relationships between statements:** Cota et al. [35] provide a method for validating the content of medical procedure documents. This is accomplished in two main steps. The medical procedure is modelized then verified using a use case graph. The latter is then transformed into a graph of relations between the various tasks of the procedure. Afterwards, the anomalies are detected by experts when analyzing the result graphs or when extracting the execution order of the tasks. These different analyses are manual. This means that the proposal can only be applied to small bases. Blanchette et al. [36] present IsaFol, a proposed formalism for the development of formal theories on logic in proof systems. The authors indicate that this formalization helps to follow the assumptions and relationships in a precise way. This makes it possible to detect anomalies in the analyzed strategy.

The works we studied suggest techniques for resolving anomalies in tiny strategies. With an increase in the size of the treated strategies, the performance of these methods and those relying on the Closure’s calculation degrades. On the other hand, other works are based from the start on the transformation of the knowledge base into a problem or a set of mathematical formulas, while others aim to rely on the semantics expressed by the statements. We built our approach on the second category of methods since semantics assists in the better identification and resolution of anomalies.

## Methods

In smart healthcare systems, devices are controlled by healthcare strategies. These may include some anomalies that cause erroneous decisions that negatively affect the patient’s health.

**Fig. 6:**
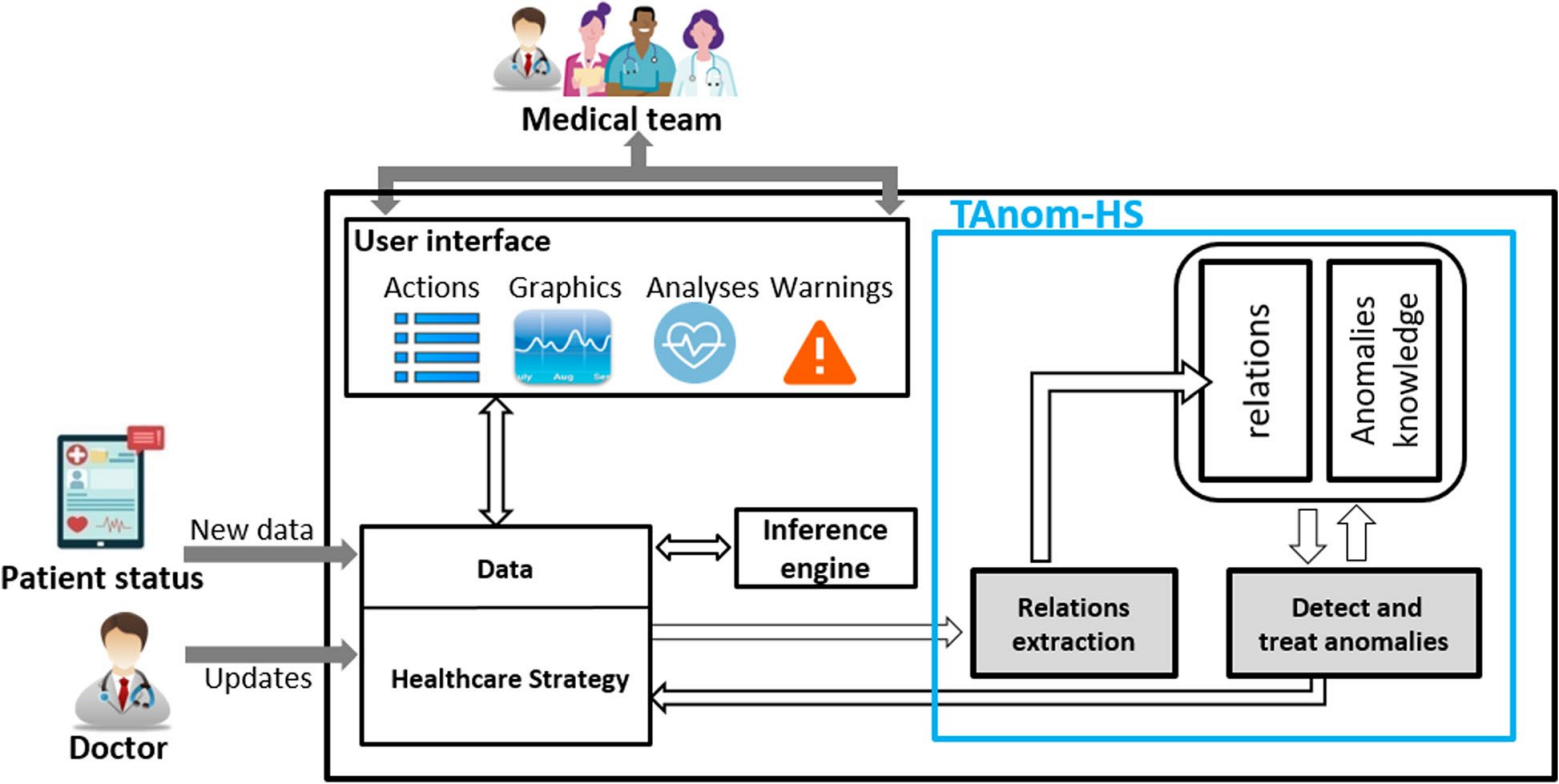
Integrating TAnom-HS in a smart healthcare system

**Fig. 7:**
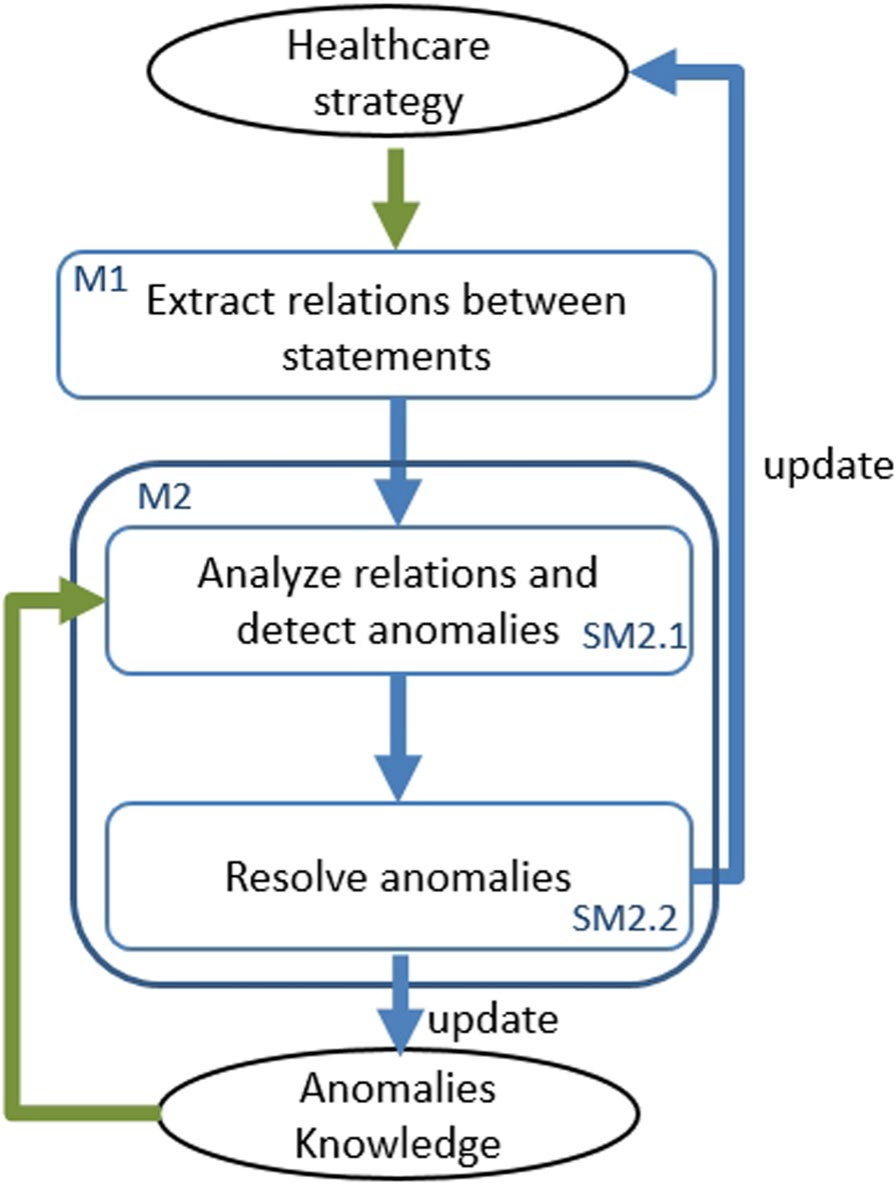
TAnom-HS approach

TAnom-HS is an approach proposed to be injected into a smart healthcare system in order to resolve anomalies (see Fig. 6). TAnom-HS performance acts in three main steps : (i) detecting the relationships between statements in the healthcare strategy, (ii) analyzing the relationships and detecting anomalies and (iii) resolving the detected anomalies. These steps are performed by two modules M1 and M2 (see Fig. 7).

### Extracting relations between statements

**Fig. 8:**
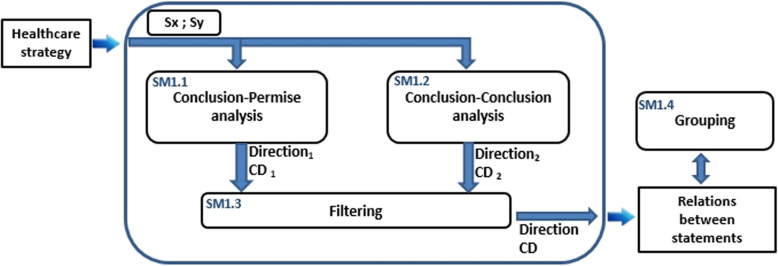
M1 module performance: relations extraction

The analysis of the relationships between the elements of a strategy allows to verify strategies.. The main objective of the M1 module is to extract the relations between the different elements of a healthcare strategy. Its general architecture is provided in Fig. 8.

**Fig. 9:**
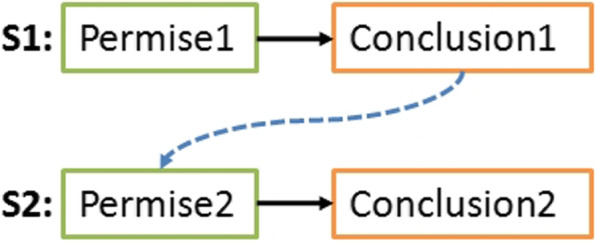
Conclusion-Premise analysis (SM1.1)

**Fig. 10:**
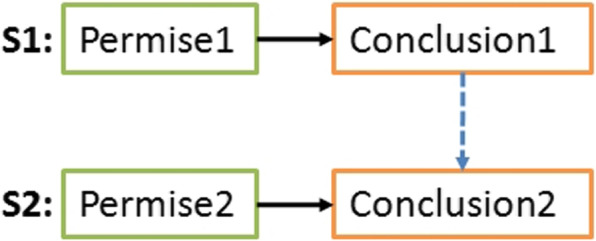
Conclusion-Conclusion technique (SM1.2)

When extracting relations between statements, at each iteration, M1 module processes a pair of statements (Sx,Sy) from the healthcare strategy. This pair goes through the first two sub-modules SM1.1 and SM1.2 for the analysis of the relation that can exist between the statement Sx and the statement Sy. Each of these sub-modules uses a different technique to provide a confidence degree $CD_{i}$, indicating the degree of certainty of the extracted relation, and a direction $direction_{i}$ indicating the direction of the relation ($D_{Sx-Sy}$ or $D_{Sy-Sx}$). The first technique is based on the analysis of the relation between the conclusion part of one statement and the premise part of the other (See Fig. 9). The second technique is based on the analysis of the relations between the facts inferred by the statements conclusions (See Fig. 10).

The pairs ($direction_{i}$, $CD_{i}$) returned by each of the two sub-modules SM1.1 and SM1.2 represent the inputs of the Filtering sub-module (SM1.3) which will retain the pair having the most appropriate values for this relationship.

#### First technique: conclusion - premise analysis

This technique allows extracting a relationship between two statements Sx and Sy based on the fact that the knowledge inferred by Sx will be used by Sy. This indicates that this module analyzes the relationship between the conclusion of the first statement, which will infer the new knowledge, and the second’s premise, which will use it (See Fig. 9).

The general algorithm of this analysis can be summed up in the following steps: (i) calculate the confidence degree of the relationship $R_{Sx-Sy}$ from Sx to Sy, (ii) calculate the confidence degree of $R_{Sy-Sx}$ the relationship from Sy to Sx, and (iii), keep the relationship with the highest confidence degree.

Calculating Confidence Degree: The SM1.1 submodule is based on looking for relationships between the classes concerned by the facts inferred by the first rule and those exploited by the second. It analyses pairs of atoms from the first statement’s conclusion and the second’s premise. It seeks the relations between the ontology’s classes and properties shared by both statements parts. Let’s take as an example computing the weight attributed to the couple of atoms (*ai*, *aj*) where $ai=Person(p1)$, an atom of the conclusion of a statement Sx, and $aj=hasCar(p,c)$, an atom of the premise of a statement Sy. Atom *ai* is a class type atom and *aj* is a data property type atom. In this case, SM1.1 starts by extracting the set of cl_si classes referenced in *ai* , and the set of cl_sj classes referenced in *aj* . It then obtains $cl\_si =\lbrace Person\rbrace$ and $cl\_sj =\lbrace Person,Car\rbrace$. There is an equality relation between the pair of classes (Person,Person), then the weight is 1. There is neither equality, nor equivalence, nor hierarchy relationship between the pair of classes (Person,Car). Then the weight is null. Keeping the highest value, the weight of atoms (ai,aj) is 1.

The SM1.1 module continues to analyze the pairs of atoms and keeps the highest weight as degree of confidence from Sx to Sy.

#### Second technique: conclusion - conclusion analysis

This technique is different from the one used before. As mentioned before, each statement takes the form of an implication between a premise and a conclusion parts. The main idea of this technique is based on the search and the analysis of relations defined between the facts inferred by a statement and the facts inferred by the other (see Fig. 10).

All inferred facts are stated in the conclusion. To measure the confidence degree of a relationship between a statement Sx and a statement Sy, this technique is based on the search and the analysis of the relations *pi* between the objects referenced by the conclusion of Sx and those referenced by the conclusion of Sy. The analysis of all these relations *pi* makes it possible to determine the direction $direction_{i}$ and the confidence degree $CD_{i}$ of the relation between the statements Sx and Sy [37].

#### Filtering and grouping extracted relations

After analyzing the relation between two statements Sx and Sy, the preceding sub-modules SM1.1 and SM1.2 provide a dependency relation that may be different from the other. The role of the Filtering sub-module SM1.3 is to keep the most appropriate dependency relation among those provided by SM1.1 and SM1.2.

This choice is made by taking into account the degree of confidence *CDi* of each dependency and the sub-module that generated it. Thus, before making its choice, the Filtering sub-module multiplies each degree of confidence *CDi* by a coefficient $c_i$ that we have already fixed by achieving some experiences. The highest coefficient (c2 = 1) is assigned to the sub-module SM1.2 (using the Conclusion-Conclusion analysis technique) because it is based on the knowledge extracted from the strategy and those extracted from the ontology. The other sub-module is based on knowledge extracted only from the strategy. Then its coefficient is lower (c1 = 0.5). The chosen relation, defined by its degree of confidence *CD* and its direction *direction*, is that which corresponds to the the highest product $DC_{i} * c_{i}$.

Most strategies can be divided into groups of statements each of which is responsible for accomplishing a specific task. As example we cite the strategy of FHHO which includes a statements group to determine the relationships between family members. It also includes another group whose elements are responsible for giving information about a person’s health. On the other hand, there are strategies where each task can only be accomplished by one statement. We cite the AO’s^3^ strategy [38] and the ADAR’s^4^ strategy. Their elements define how each phenotype should be derived from a set of clinical outcomes. To do this, each statement is responsible for inferring new knowledge without using any other statement.

In TAnom-HS (Fig. 8), the M1 module collects statements, using Grouping sub-module (SM1.4), in order to facilitate and optimize the task of resolving anomalies. This sub-module is based on the relations already extracted. The statements of the same group take as input facts that relate to the same objects. They also produce facts that concern the same objects. Thus, the statements of the same group have the same incoming arcs and the same outgoing arcs.

### Resolve anomalies in Healthcare strategies

**Fig. 11:**
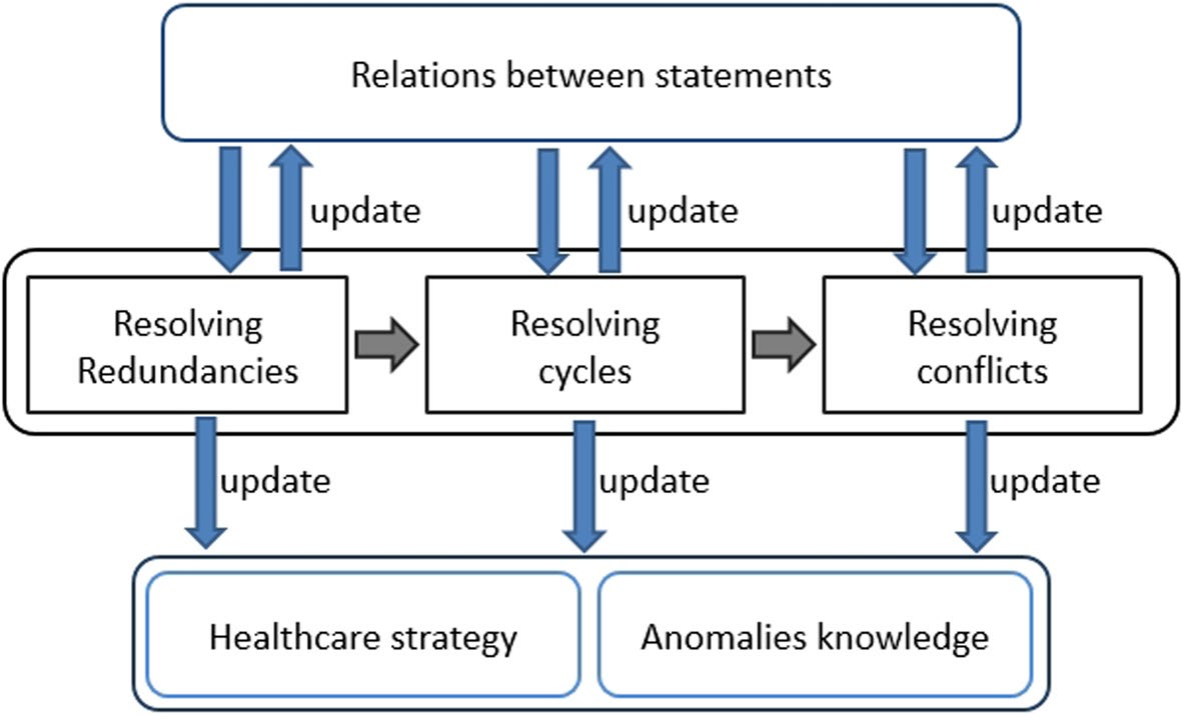
M2 module performance : resolving anomalies

The task of this module is to eliminate anomalies that may affect a strategy. It is based on the relations already extracted by the previous module. It is also based on groups of rules already built. This will reduce the number of iterations when processing anomalies. The general architecture of the strategy anomalies resolution module is shown in Fig. 11. We started by removing any type of redundancy to optimize the subsequent handling of anomalies.

#### Resolving redundancies

Redundancy identifies criteria or inferences that can be ignored [36]. It contributes to the unnecessary increase in the size of a strategy. There are three types of redundancies: equivalence, subsumption, and transitivity.

##### Resolving equivalence

Equivalent statements have the same inputs and outputs. This explains their belonging to the same groups built by the grouping sub-module (SM1.4). Thus, the search for equivalent statements can be done within each group *Gi* to reduce the number of iterations. Instead of checking the equality of a statement with all the other ones of the strategy, it is enough to compare it to the statements of the group to which it belongs.

In literature, equivalence is detected due the use of the same data. In our work, we add the possibility of relying on hierarchical relationships. Thus, we define equivalence based on the relation graph as:

*Equivalence* - Two statements Sx, Sy are equivalent if and only if :

- $${Sx, Sy} \in$$*Gi*^5^
- Sx and Sy have corresponding premises^6^ and corresponding conclusions.

All cases we considered are presented using Example 4. The details and the solutions for their elimination are presented in the following paragraph.

***Example 4.***

$${\textbf {S01:}} Patient(x), hasFood(x,y), hasIngredient(y,z) \longrightarrow eats(x,z)$$

$${\textbf {S02:}} Patient(x), hasIngredient(y,z), hasFood(x,y) \longrightarrow eats(x,z)$$

$${\textbf {S03:}} Patient(x), hasLunch(x,y), hasIngredient(y,z) \longrightarrow eats(x,z)$$

$${\textbf {S04: }}Patient(x), hasFood(x,y), hasIngredient(y,z) \longrightarrow gets(x,z)$$

**Fig. 12:**
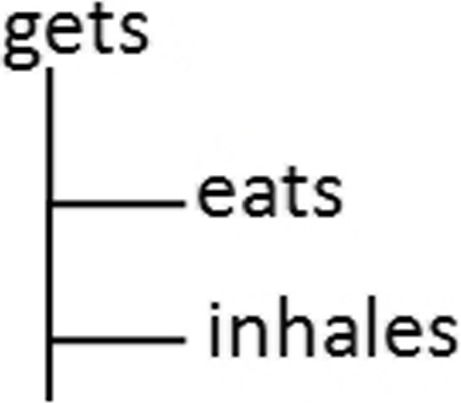
Object property tree

- **Case 1 -** Both statements have the same atoms that are not invoked in the same order (such as S01 and S02). In this case we eliminate one of these two statements.
- **Case 2 -** The parts of the statements correspond. Equivalence redundancy is caused by hierarchical relationships between two atoms a1 and a2. Here, we distinguish two sub-cases:
  - **Case 2.1:** If *a1* is an atom of the premise of the first statement and *a2* is an atom of the conclusion of the second one: eliminating this redundancy is done by deleting the statement including the atom which refers to the most elementary entity (the lowest in the hierarchy in the ontology definition). In Example 4, statements S01 and S03 are equivalent. Note the difference caused by *a1*=hasFood(x,y) in the premise of S01 and *a2*=hasLunch(x,y) in the premise of S03. *a1* references the relation p1=hasFood(Patient, Food) and *a2* references the relation p2=hasLunch(Patient,Lunch). Relation p2 is defined in the ontology as a sub-relation of p1. Thus, the facts valid for p2 are also valid for p1, while the opposite case is not always true. It is obvious then that the deletion of rule R03 is the most adequate solution for this case of redundancy.
  - **Case 2.2:** If *a1* is an atom in the first statement’s conclusion and *a2* is an atom in the second’s conclusion : we solve this case of redundancy by eliminating the statement which includes the atom referencing the parent entity (the highest in the hierarchy defined by ontology). In Example 4, statements S01 and S04 are equivalent. The difference lies in atoms *a1*=eats(x,z) and *a2*=gets(x,z) in their conclusions. *a1* refers to the relation p1=eats(Patient,Food) and *a2* refers to the relation p2=gets(Patient,Edible). Since p1 is a parent property of p2 (see Fig. 12), then the facts inferred by S01 are also valid for the facts inferred by S04, whereas the opposite case is not true. Therefore, deleting S01 can cause loss of knowledge during the inference process. So S04 is the one to be eliminated.

##### Resolving subsumption

Two statements Sx and Sy have a subsumption relationship if they share the same conclusion while the premise of one subsumes the other (in Example 5, S06 subsumes S05).

***Example 5.***

$${\textbf {S05:}} {\textbf {hasFood(x,y)}}, hasIngredient(y,z) \longrightarrow eats(x,z)$$

$${\textbf {S06:}} Patient(x), Food(y), hasFood(x,y), hasIngredient(y,z) \longrightarrow eats(x,z)$$

$${\textbf {S07:}} {\textbf {hasLunch(x,y)}}, hasIngredient(y,z) \longrightarrow eats(x,z)$$

$${\textbf {S08: }}{} {\textbf {hasBreakfast(x,y)}}, hasIngredient(y,z) \longrightarrow eats(x,z)$$

$${\textbf {S09:}} {\textbf {hasDiner(x,y)}}, hasIngredient(y,z) \longrightarrow eats(x,z)$$

We distinguish two cases of subsumption:

- **Case 1 : simple subsumption:** both statements share the same conclusion, but the premise of the first is a part of the premise of the other (Example 5: S06 subsumes S05. S06 is the statement to delete).
- **Case 2 : semantic subsumption:** there are a general statement and some statements which present particular cases giving all the same result (Example 5: S07, S08 and S09 are particular cases of S05. So we keep S05 and delete S07, S08 and S09).

To detect subsumption redundancies, we propose to start by analyzing the incoming arcs and the outgoing arcs of the statement groups. Then, relations of subsumption are analyzed :

- between the couples of statements within the same group (Example 5 - S05 and S06 : object *Patient* belongs to the participating objects in the premise of S05)
- and between the couples of statements Si, Sj such as:
  - $$Ri \in Gi$$
  - $$Rj \in Gi$$
  - $$In(Gi) \subset In(Gj)$$^7^
  - $$Out(Gi) = Out(Gj)$$^8^

Using statements groups allows reducing the iterations number during the analysis of the subsumption relations between the statements. Thus, instead of treating all pairs of statements (Sx, Sy) in the strategy, we deal only with those that belong to the same group, and those belonging to the groups that satisfy the criteria that we have just mentioned.

##### Resolving transitivity

Transitivity exists if a statement can be inferred from two or more other statements. Thus, a transitive statement must be deleted. In Example 6, S12 can be deduced from S10 and S11. S12 is said to be redundant for the strategy and it must be deleted.

***Example 6.***

$${\textbf {S10:}} Person(x), hasFBST(x,i), equalOrGreaterThan(i,1.26), hasSymptomsT2(x,True) \longrightarrow DiabetesType2(x)$$

$${\textbf {S11:}} DiabetesType2(x) \longrightarrow gets(x, Metformin)$$

$${\textbf {S12:}} Person(x), hasFBST(x,i), equalOrGreaterThan(i,1.26) hasSymptomsT2(x,True) \longrightarrow gets(x, Metformin)$$

Using the relations already extracted between statements, we define a transitive statement St by the fact that it can be replaced by a tuple CH=$\langle Sa,Sb,..,Sn\rangle$ composed of other statements. This implies that :

- St uses in its premise all the data used in the premises of the statements Si of the tuple CH
- St has the same conclusion as, or an equivalent conclusion to, that of Sn (the last statement of the tuple CH).

**Algorithm 1:**
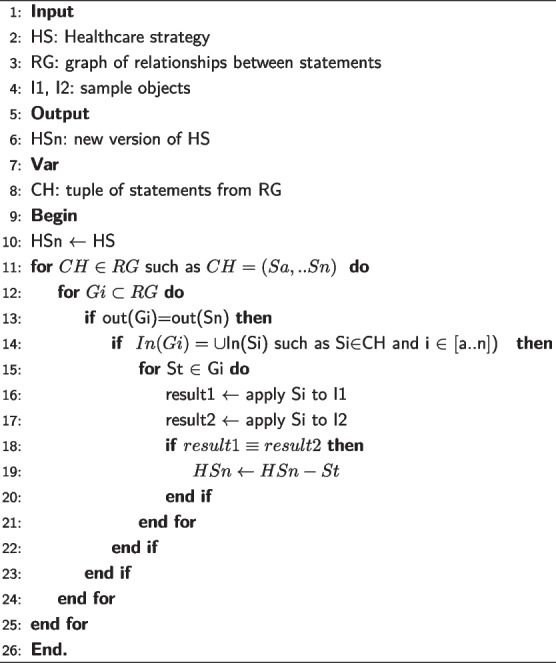
Dealing with transitive statements

**Definition 1. transitivity.**

A statement St is said to be transitive if and only if, in the graph of relations there exists a tuple CH=$\langle Sa,Sb,..,Sn\rangle$ such as:

- *In*(*St*) = $$\bigcup _{i=0}^{n} In(Si)$$
- $$Conclusion (St) \equiv Conclusion (Sn)$$

The process we followed for the detection of transitive statements is formulated by Algorithm 1. To minimize the number of iterations, we relied on the statements’ groups. We then compared the set of outgoing arcs of each group *Gi* to the set of outgoing arcs of each tuple CH in the relationship graph (Algorithm 1, L13). We also compared the set of incoming arcs to the statements that make up CH to the set of incoming arcs to the *Gi* group (Algorithm 1, L14). Equality between sets of incoming arcs and equality between sets of outgoing arcs indicates a possibility of finding transitive statements. So we check each statement St in the group *Gi* (Algorithm 1, L15). To do this, we apply CH and each statement St to some examples of objects (Algorithm 1, L16-L17). Obtaining the same result implies that St can be replaced by CH. Then St is transitive and must be eliminated (Algorithm 1, L18-L20).

#### Resolving cycles

Analysing cycles in some relations graphs, we noticed that there are fake cycles (See Example 7). We proposed to save them in *anomalies knowledge* in order to consider them during the processing of the next cycle. On the other hand, the real cycles are also saved in the same knowledge base in order to expose them to the experts for a possible resolution and in order to avoid them during the next inferences.

***Example 7.*** fake cycle

The following statements relate to drugs with the same category and equivalent drugs.

$${\textbf {St01:}} Drug(d1), {\textbf {hasCategory}}(d1,c) , hasEquivalent(d1,d2) \longrightarrow {\textbf {hasCategory}}(d2,c)$$  

$${\textbf {St02:}} {\textbf {hasCategory}}(d2,c) \longrightarrow {\textbf {includes}}(c,d2)$$

$${\textbf {St03:}} Drug(d2), {\textbf {includes}}(c,d2) , Drug(d3), hasEquivalent(d3,d2) \longrightarrow {\textbf {hasCategory}}(d3,c)$$

These statements form a cycle in the relations graph ($\langle St01, St02, ST03 \rangle$). We see that the cycle does not actually exist when applied to instances **d1** and** d2** and *d3* of the Drug class and **c** instance of the Category class.

#### Resolving conflicts

In literature, statements are said to be conflicting if they take the same input and return conflicting results [39]. We believe that some cases of contradictions may have been missed when dealing with anomalies as some cases of statements may have contradictions in their premises. These contradictions, in some cases, cause conflicts during the inference process (see Example 8). In our work, we consider two statements as conflicting ones if they have two corresponding premises (resp. conclusions) and two contradictory or partially contradictory conclusions (resp. premises).

***Example 8.*** conflicts in the premises of two statements

$${\textbf {S10:}} Person(x), hasFBST(x,i), {\textbf {equalOrGreaterThan}}(i,1.26) hasSymptomsT2(x,True) \longrightarrow DiabetesT2(x)$$

$${\textbf {S13:}} Person(x), hasFBST(x,i), {\textbf {equalOrLessThan}}(i,1.26) hasSymptomsT2(x,True) \longrightarrow DiabetesT2(x)$$

**Definition 2. Contradictory premises - Contradictory conclusions.**

Consider two statements Sx and Sy. We say that Premise(Sx) and Premise(Sy) (respectively Conclusion(Sx) and Conclusion(Sy)) are contradictory if they express conflicting facts.

**Definition 3. Partially contradictory premises - partially contradictory conclusions.**

Consider two statements Sx and Sy. We say that Premise(Sx) and Premise(Sy) (respectively Conclusion(Sx) and Conclusion(Sy)) are partially contradictory if there are literals^9^ p1x, p2x, p1y and p2y such as :

- **Case 1:**
  - Premise (Sx) = $$p1x \cup p2x$$
  - Premise (Sy) = $$p1y \cup p2y$$
  - p1x and p1y correspond
  - p2x and p2y are contradictory
- **Case 2:**
  - Conclusion (Sx) =$$p1x \cup p2x$$
  - Conclusion (Sy) = $$p1y \cup p2y$$
  - p1x and p1y correspond
  - p2x and p2y are contradictory

**Definition 4. contradictory literals.**

Let l1 and l2 be two literals. l1 and l2 are said to be contradictory if they express conflicting facts.

***Example 9.***

In the following case, literals 11 and 12 are contradictory.

l1=hasFBST(x,i), equalOrGreaterThan(i,1.26), hasSymptomsT2(x,True)

l2=DiabetesType1(x).

We distinguish two types of conflicts: strong conflicts and probable conflicts. A conflict is called strong when there is no doubt that the analyzed statements are conflicting. A conflict is said probable if the analyzed statements may be non-conflict. In this case, it is imperative to take the medical team’s opinion.

**Strong conflict :** Two statements are strongly conflicting if their premises correspond and their conclusions are contradictory (see Example 10).

***Example 10. Strong conflict.***

Two statements Sx and Sy with the following forms are strongly conflicting (such as **S10** and **S14**)

$${\textbf {Sx:}} a \longrightarrow b$$

$${\textbf {Sy: }}a \longrightarrow \lnot b$$

$${\textbf {S10:}} Person(x), hasFBST(x,i), equalOrGreaterThan(i,1.26) hasSymptomsT2(x,True) \longrightarrow {\textbf {DiabetesT2(x)}}$$

$${\textbf {S14:}} Person(x), hasFBST(x,i), equalOrGreaterThan(i,1.26) hasSymptomsT2(x,True) \longrightarrow {\textbf {DiabetesT1(x)}}$$

**Probable conflict :** Two statements Sx and Sy are probably conflicting if they occur in one of the following cases:

- **Case 1:** Their premises are contradictory, whereas their conclusions correspond (see Example 11).
  ***Example 11.*** Probable Conflict - Case 1 Two statements Sx and Sy having the following forms (such as **S11** and **S15**):
  $${\textbf {Sx:}} a \longrightarrow b$$
  $${\textbf {Sy:}} \lnot a \longrightarrow b$$  
  $${\textbf {S11:}} {\textbf {DiabetesType2}}(x) \longrightarrow gets(x, Metformin)$$
  $${\textbf {S15:}} {\textbf {DiabetesType1}}(x) \longrightarrow gets(x, Metformin)$$
- **Case 2:** The conclusions of the statements correspond while literals of their premises are contradictory (see Example 12).
  ***Example 12.*** Probable Conflict - Case 2 Two statements Sx and Sy having the following forms (such as **S10** and **S13**):
$${\textbf {Sx:}} a, b \longrightarrow c$$
$${\textbf {Sy:}} a, \lnot b \longrightarrow c$$
  $${\textbf {S10:}} Person(x), hasGI(x,i), {\textbf {equalOrGreaterThan(i,1.26)}} \longrightarrow DiabetesT2(x)$$
  $${\textbf {S13:}} Person(x), hasGI(x,i), {\textbf {equalOrLessThan(i,1.26)}} \longrightarrow DiabetesT2(x)$$

The main purpose of probable conflicting statements is to warn the experts of possibly existing conflicts. The probable conflicts are saved and reported to the experts who can validate them and show how they can be solved. If the conflict is resolved, it is removed from the *anomalies knowledge* base. Otherwise, it is saved as a false conflict for consideration in further verification.

While resolving conflicts, TAnom-HS uses the graph of already extracted relations by analyzing the incoming and outgoing arcs of each group of statements. Take the example of two strong conflicting statements. These have the same incoming arcs. Thus, it is sufficient to check if the conclusions of these statements are contradictory. So, we optimize the conflict-handling process by reducing the number of analyzed statements.

#### Resolving inaccessible statements

Inaccessible statements are statements whose premise, or conclusion, cannot be satisfied. This is due to the presence of unsatisfactory literals (see Example 13).

­***Example 13.*** Non-satisfying literals

p1, p2 and p3 are non-satisfying literals.

p1 = DiabetesType1 and p2 = DiabetesType2

p1 = DiabetesType1 and p3 = hasFBST(x,i), equalOrGreaterThan(i,1.26), hasSymptomsT2(x,True)

**Algorithm 2:**
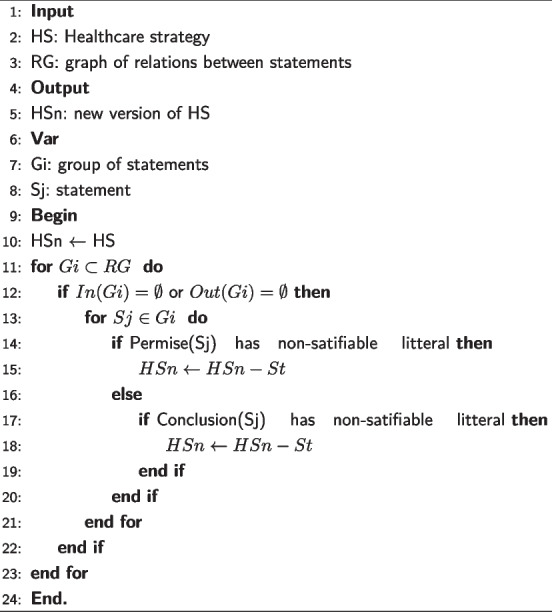
Dealing with inaccessible statements

In a graph of relations between statements, an inaccessible statement is a statement represented by a node with no incoming arcs or outgoing arcs. This condition is necessary but not sufficient. Note the example of a statement charged of a task and that does not depend on any other statement, and the example of a statement that is at the end of the inference process.

Detecting and resolving anomalies module in TAnom-HS processes inaccessible statements according to Algorithm 2. Thus, it looks for the groups of statements having no incoming arcs or no outgoing arcs or, having neither incoming arcs nor outgoing arcs (Algorithm 2, L12). Group’s arc analysis can be used to optimize the process of handling inaccessible statements. Within each group, the algorithm analyzes the premise and conclusion parts of each statement. It checks the non-satisfiable literals. A statement with at least one non-satisfiable literal is automatically eliminated (Algorithm 2, L14-L20).

## Results

In order to avoid making decisions that are inappropriate for the case being treated, it is necessary to treat anomalies in medical strategies. To achieve this, we developed TAnom-HS, which produced the results presented in this section.

To evaluate TAnom-HS, we implemented a prototype named HS-check integrated into Protégé editor^10^. We used three use cases from real life (see Table 2). CDPEO^11^ and OntoFood^12^, and PCMO^13^ were selected from the Bioportal. We draw attention to the rarity of strategies that come with their proper execution orders. So we selected the strategies that we were able to access their different execution scenarios in order to be able to conduct our evaluation.

### Evaluation of relations extraction

**Fig. 13:**
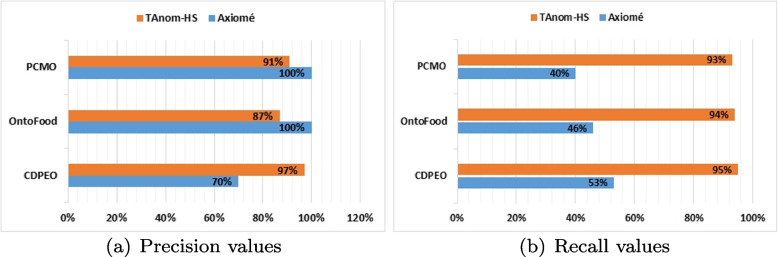
Experiments results

As presented before, our approach acts in two main steps: extracting relations and handling anomalies. In this section, we present the evaluation of our proposal for relations extraction. To position ourselves to existing work on relationships extraction, we compared M1 module to Axiomé [13]. Axiomé is a method based on the most complete atom analysis. It is available as a plugin for Protégé. We applied Axiomé and all M1’s sub-modules on the selected study cases. We present the results obtained using Fig. 13. These values were calculated by comparing the obtained relations to those provided by strategies developers. Figure 13a shows the rates of precision and Fig. 13b shows the rates of recall in each case. Precision and recall are a sensitivity measurement expressing how well the approach is predicting the true positives^14^ compared to the number of false negatives^15^ and false positives^16^.

So they are computed as following :

$$Precision = true~positives / (true~positive + false~postives)$$

$$Recall = true~positives / (true~positive + false~negatives)$$

### Evaluation of resolving anomalies

In this section, we present the evaluation of our proposal for handling anomalies. The ontologies we considered are directly provided by their developers and have not been changed yet. So, their strategies are consistent and they do not contain redundant statements nor cycles, or conflicting rules. And to evaluate the performance of Tanom-HS, we have made some random changes to the statements bases. We have tried to put, in each study case, all the types of anomalies resolved taking into account all the cases of each of these anomalies. In Table 2 we present data about the state of each statements base. We applied our proposal to each one, and we compared the results with the initial states.

**Table 2:** Statements bases state before and after random changes

	CDPEO	OntoFood	PCMO
# classes	40	292	24
# properties	98	48	12
# stmts (initial state)	80	24	94
# added redundant stmts	+22	+8	+17
# added stmts causing cycles	+6	+4	+5
# added stmts causing conflicts	+14	+8	+12
# inaccessible stmts	+8	+3	+6

#### Resolving redundancy -

To verify the redundancy removal sub-module, we manually determined the redundancy cases that existed before and after its application. This task is based on the list of added statements which is already known. We then obtained the results presented in Table 3. This latter shows that TAnom-HS has eliminated all the redundant statements from the second and third study cases. We checked the deleted statements and the new state of the statements base.

**Table 3:** Results of resolving redundancy

	**study case 1**	**study case 2**	**study case 3**
# redundant stmt pairs before TAnom-HS	22	8	17
# redundant stmt pairs after TAnom-HS	2	0	0

#### Resolving cycles -

During this evaluation, we were interested in finding simple cycles do this, we relied on the taboo search algorithm [40] often used for finding paths in a graph. We edited this algorithm for cycle search by checking if the starting statement is the ending one. This algorithm allows to extract all the paths in a graph. The results of this experiment are presented in Table 4.

**Table 4:** Results of resolving cycles

	**study case 1**	**study case 2**	**study case 3**
# cycles before TAnom-HS	68	36	54
# correct cycles saved by TAnom-HS	43	12	34
# fake cycles saved by TAnom-HS	25	24	20

#### Resolving conflicts -

After resolving conflicts, we consulted the statements bases to verify the statements causing conflicts. We also verified the *anomalies knowledge base* to see the list of saved conflicts (an example of a saved conflict is presented in Example 14).

***Example 14. detected conflicting statements***

**S22:** Food(f) , has-glycemic-index(f,gi) , lessThanOrEqual(gi,70)

$\longrightarrow$ has-meal-level(f,high-level)

**S22C:** Food(f) , has-glycemic-index(f,gi) , lessThanOrEqual(gi,70)

$\longrightarrow$ has-meal-level(f,very-low-level)

$\triangleright$ Such a conflict is saved in the anomalies knowledge base for experts’ opinion.

The results we obtained are shown in Table 5.

**Table 5:** Handling conflicts results

	**study case 1**	**study case 2**	**study case 3**
# conflict stmt pairs before TAnom-HS	14	8	12
# new stmts deleted by TAnom-HS	2	3	2
# old stmts deleted by TAnom-HS	1	2	2
# conflict stmt pairs saved by TAnom-HS	11	3	8

#### Handling inaccessible statements -

To assess this type of anomaly, we have added a set of statements with contradictory facts in their premises or in their conclusions. After applying our proposal to the study cases, we compared the statements bases provided by their developers to those provided by our proposal.

## Discussion

This part presents an examination of the findings from the preceding section. Analysis of the relations extractions evaluation’s findings comes first. The findings from the anomalies resolution will then be discussed.

### Relations extraction

Figure 13 shows that in the first case, Axiomé extracted a small number of relations among which nearly 57% are false (precision = 43%). Whereas TAnom-HS has practically extracted all the relations requested with almost perfect precision (equal to 97%). These results are due to the fact that Axiomé only considers a few types of atoms, while 54% of the statements in the second case are based on these unconsidered atoms. In the second and third cases, the statements include only atoms of types considered by Axiomé. Thus, Axiomé did not extract false relationships. It managed to extract almost half of the number of requested relations. As for TAnom-HS, it extracted almost all the requested relations with a low number of false relations. This improvement in results is due to the fact that our proposal involves the technique used by Axiomé and extends it with other techniques, as previously indicated.

In addition, in the statements base of the first case, the relationship graph generated by Axiomé includes 62 isolated rules (which have neither incoming nor outgoing arcs). But the graph generated by TAnom-HS does not include any isolated rule. This is conform with the results provided by the developers of the study cases. We can therefore conclude that TAnom-HS has improved the results of extracting relationships between statements compared to existing work.

### Resolving anomalies

#### Resolving redundancy -

Referring to Table 3, we did not find any deleted statements other than redundant ones. Besides, this table indicates that there are 2 redundant statements left. This is due to some atoms describing the same relation using different expressions. The following example is one of these cases.

***Example 15.***

**R08:** PatientProfile(p), hasSmoking(p,s), hasDailyCigarettes(s,d),

**greaterThanOrEqual**(d,21)$$\longrightarrow$$ vectorItemSmoking(p,3)

**R08b:** PatientProfile(p), hasSmoking(p,s), hasDailyCigarettes(s,d),

**moreThan**(d,20)$$\longrightarrow$$ vectorItemSmoking(p,3)

#### Resolving cycles -

Table 4 shows that TAnom-HS has succeeded in detecting all cycles.

The correct cycles are saved in the *anomalies knowledge* base so that they can be consulted by the experts for a possible resolution. In addition, during inference processes, inference engines can also avoid them. This allows ensuring that the system does not fall into a state of blocking due to anomalies in the statements base.

#### Resolving conflicts -

Table 5 shows that all conflicting statements are detected. But we noticed that some conflict resolutions caused the loss of statements defined by the developers of the base. Our proposal is not totally flawed, as it allowed us to eliminate a large number of statements that caused conflicts (Table 5, Lines 3 and 4). The table also shows that all conflict cases are detected and dealt with.

#### Handling inaccessible statements -

Comparing the statements bases provided by their developers to those provided by our proposal, we noticed that all the inaccessible statements have been deleted except one statement in the first case study. The statement has two atoms referencing two properties, isGreaterThan(x,y) and isLessThan(x,y), which perform conflicting checks. These two properties are not defined as inverse properties in the database.

## Conclusion

Statements supporting intelligent medical systems’ performance are continuously modified to be up to date with new states. These changes may cause inevitable anomalies which have to be eliminated to ensure information consistency and service quality. In this paper, we proposed a method called TAnom-HS to verify and optimize statement bases. We defined anomalies using relations between statements and we presented solutions for their resolution. The evaluation results are promising. We plan to improve our work and address additional forms of anomalies that may emerge in the knowledge base. We also intend to study works based on machine learning technologies to improve the quality of decision-making.

## Data Availability

CDPO strategy available at bioportal.bioontology.org/ontologies/CDPEO. Ontofood strategy available at bioportal.bioontology.org/ontologies/OF. PCMO strategy is available at https://bioportal.bioontology.org/ontologies/PCMO.

## Abbreviations

ADAR: Autism DSM-ADI-R
AO: Autism Ontology
BRMS: Business Rules Management Systems
CDPO: Chronic Disease Patient Education Ontology
FHHO: Family Health History Ontology
OWL-S: Web Ontology Language for Services
PCMO: Pediatric Consultation and Monitoring Ontology
SWRL: Semantic Web Rule Language
TAnom-HS: Treating Anomalies in Healthcare Systems

## Footnotes

1 Family Health History Ontology (https://bioportal.bioontology.org/ontologies/FHHO)

2 https ://www.drools.org/

3 Autism Ontology

4 Autism DSM-ADI-R ontology (https://bioportal.bioontology.org/ontologies/ADAR)

5 *Gi*: a group of statements

6 corresponding parts : parts having equal or equivalent atoms

7 *In*(*Gi*): incoming arcs to group Gi

8 *Out*(*Gi*): outgoing arcs from group Gi

9 a literal is a positive conjunction of some atoms (l=$a1 \wedge a2 \wedge ..\wedge an$)

10 https://protege.stanford.edu/

11 https://bioportal.bioontology.org/ontologies/CDPEO

12 https://bioportal.bioontology.org/ontologies/OF

13 https://bioportal.bioontology.org/ontologies/PCMO

14 True positives: number of correct relations extracted

15 False negatives: number of correct relations not extracted

16 False positives: number of false relations extracted
